# GPU accelerated sequence alignment with traceback for GATK HaplotypeCaller

**DOI:** 10.1186/s12864-019-5468-9

**Published:** 2019-04-04

**Authors:** Shanshan Ren, Nauman Ahmed, Koen Bertels, Zaid Al-Ars

**Affiliations:** Delft University of Technology, Mekelweg 4, Delft, 2628 CD The Netherlands

**Keywords:** Semi-global alignment with traceback, Optimal alignment, GATK HaplotypeCaller (HC), GPUs

## Abstract

**Background:**

Pairwise sequence alignment is widely used in many biological tools and applications. Existing GPU accelerated implementations mainly focus on calculating optimal alignment score and omit identifying the optimal alignment itself. In GATK HaplotypeCaller (HC), the semi-global pairwise sequence alignment with traceback has so far been difficult to accelerate effectively on GPUs.

**Results:**

We first analyze the characteristics of the semi-global alignment with traceback in GATK HC and then propose a new algorithm that allows for retrieving the optimal alignment efficiently on GPUs. For the first stage, we choose intra-task parallelization model to calculate the position of the optimal alignment score and the backtracking matrix. Moreover, in the first stage, our GPU implementation also records the length of consecutive matches/mismatches in addition to lengths of consecutive insertions and deletions as in the CPU-based implementation. This helps efficiently retrieve the backtracking matrix to obtain the optimal alignment in the second stage.

**Conclusions:**

Experimental results show that our alignment kernel with traceback is up to 80x and 14.14x faster than its CPU counterpart with synthetic datasets and real datasets, respectively. When integrated into GATK HC (alongside a GPU accelerated pair-HMMs forward kernel), the overall acceleration is 2.3x faster than the baseline GATK HC implementation, and 1.34x faster than the GATK HC implementation with the integrated GPU-based pair-HMMs forward algorithm. Although the methods proposed in this paper is to improve the performance of GATK HC, they can also be used in other pairwise alignments and applications.

## Background

NGS (Next Generation Sequencing) platforms offer the capacity to generate large amounts of DNA sequencing data in a short time and at a low cost. However, the analysis of the dramatic amounts of DNA sequencing data is still a computational challenge. Researchers have proposed many methods to improve the performance of the DNA sequencing data analysis tools and applications. One method is to execute these tools and applications on high performance computing architectures, such as supercomputers, clusters and even cloud environments. Another method is to use accelerators, such as GPUs and FPGAs, to accelerate the time-consuming kernels of these tools and applications to improve their performance.

GATK HaplotypeCaller (HC) is a popular variant caller, which is used to find the differences (or variants) between the sample DNA sequence compared with the reference sequence. Although GATK HC has higher accuracy of identifying variants compared with many other variant callers, its feasibility is limited by the long execution time needed for the analysis, which has proven to be difficult to optimize. This has driven researchers to improve its performance. Intel and IBM researchers both employ vector instructions to optimize the pair-HMMs forward algorithm [1, 2], which is the most time-consuming part of GATK HC, to reduce the total execution time. Ren et al. [3, 4] uses GPUs to accelerate the pair-HMMs forward algorithm in GATK HC, which achieved 1.71x speedup in single thread mode. After accelerating the pair-HMMs forward algorithm on GPUs, profiling of GATK HC shows that the semi-global pairwise sequence alignment accounts for around 34.5% of the overall execution time, making it the most time-consuming kernel in the application. This provides an opportunity to further improve the performance of GATK HC using GPU acceleration.

Pairwise sequence alignment, which includes global alignment, semi-global alignment and local alignment, is one of the commonly used techniques in many biological tools and applications. The global alignment and the semi-global alignment are calculated by the Needleman-Wunsch algorithm and the modified Needleman-Wunsch algorithm, respectively, while the local alignment is calculated by the Smith-Waterman algorithm. Although there are some differences existing in the three algorithms, the main framework of these algorithms is similar, which includes two stages: (1) a dynamic programming kernel to calculate the score matrices and find the optimal alignment score; (2) a traceback (or backtracking) kernel to find the optimal alignment.

Since three kinds of pairwise sequence alignment (global, semi-global and local) have the same framework and differ only in details, techniques of speeding up one can be applied to the other two with tiny modifications. Different kinds of high-performance platforms, especially accelerators, such as FPGAs [5, 6] and GPUs [7–16], are used to reduce their execution time.

There has been much research done to reduce the execution time of the three kinds of pairwise alignment on GPUs. There are two approaches to implement the first stage of the pairwise sequence alignment on GPUs (which is to calculate the optimal alignment score): inter-task parallelization model and intra-task parallelization model. The former is that each thread performs one alignment independently, such as [7] and [8]. The latter is that threads in a block cooperate to perform an alignment, such as [9]. If the pairwise sequence alignment is applied for sequence database scanning, aligning a query sequence with all database sequences for sequence similarity, a query profile and related data storage and access techniques are employed to reduce memory accesses on GPUs, such as [10] and [11]. In [11], alignments are performed in interleaved mode in order to amortize the cost of initiating each execution pass.

However, very few researchers implement the second stage on GPUs. The existing implementations can be classified into two groups. The implementations of the first group are based on backtracking matrices. Liu et al. [11] proposed to store the score matrices and backtrack the score matrices to obtain the optimal alignment. However, the method is not described clearly. gpu-pairAlign [12] proposed to store the alignment moves in four Boolean backtracking matrices during the first stage and retrieve the four Boolean backtracking matrices instead of the score matrices. This group of implementations obtain the optimal alignment in linear time, but the disadvantage is that their space complexity is quadratic. The implementations of the second group are based on the Myers-Miller algorithm. MSA-CUDA [13] developed a stack-based iterative implementation of the Myers-Miller algorithm [17] to retrieve the optimal alignment in linear space. SW# [14] proposed a modified Myers-Miller algorithm. CUDAlign 2.0 [15] combined the Myers-Miller and Smith-Waterman algorithm. Moreover, with several versions of incremental optimizations, CUDAlign 4.0 [16] is able to achieve the optimal alignment of chromosome-wide sequences using multiple GPUs. However, their approaches have quadratic time complexity, making them only suitable for the pairwise alignment of very long DNA and protein sequences.

In this paper, we provide an accelerated solution tailored to GATK HC which implements the semi-global pairwise sequence alignment with traceback on GPUs to further improve the performance. The contributions of this paper are as follows:

- We first analyze the characteristics of the semi-global alignment in GATK HC and then propose a GPU-based implementation of the semi-global alignment with traceback based on the analysis.
- During the first stage, we propose to record the length of consecutive match(es)/mismatch(es) and store the alignment moves in a special backtracking matrix.
- We also propose a new algorithm that allows for retrieving the optimal alignment efficiently on GPUs.
- We benchmark the results and show an overall speedup of GATK HC of about 2.3x over the non-accelerated version.

Although this paper proposes to improve the performance of GATK HC, the GPU-based implementation of the semi-global alignment with traceback can be used in other applications and tools. Moreover, since there are only small differences among the global alignment, semi-global alignment and local alignment, the methods proposed in this paper can also be applied to the global alignment and local alignment.

## Methods

### A brief overview of semi-global alignment

**Fig. 1:**
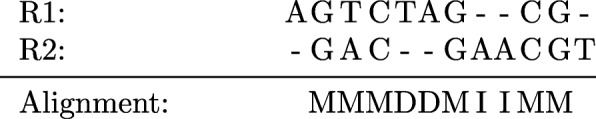
An example of an semi-global alignment of two sequences in GATK HC. R1 and R2 represent two sequences. Gaps (‘-’) at the start and end of two sequences are neglected. In the alignment, there are three kinds of operations indicating how R2 aligns with R1. ‘M’ indicates that a base in R2 aligns with a base in R1 (matches or mismatches); ‘I’ indicates that a base in R2 is not in R1; ‘D’ indicates that a base in R1 is not in R2

Semi-global alignment finds the overlap between two sequences. Insertion and deletions introduce gaps in the alignment. Gaps at the start or end of the sequences may be neglected. Hence, different types of semi-global alignments are possible between two sequences. Figure 1 shows an example of the type of the semi-global alignment performed in GATK HC.

The pairwise sequence alignment is to find the optimal alignment between two sequences, which has the optimal alignment score. The modified Needleman-Wunsch algorithm with affine gap penalties to calculate the optimal alignment score of the semi-global alignment in GATK HC is defined as

Initialization:

1 $$ \begin{aligned} M_{i,0} &=0 & (0\leq i\leq m)\\ M_{0,j} &=0 & (0\leq j\leq n)\\ \end{aligned}  $$

Recurrence:

2 $$ \begin{aligned} M_{i,j} & = max \left\{ \begin{array}{l} M_{i-1,j-1} + sbt\left({R1[i],R2\left[j\right]} \right) \\ D_{i,j} \\ I_{i,j} \\ \end{array} \right.\\ D_{i,j} & = max \left\{ \begin{array}{l} D_{i-1,j}- \beta \\ M_{i-1,j}- \alpha \\ \end{array} \right.\\ I_{i,j} & = max \left\{ \begin{array}{l} I_{i,j-1}-\beta \\ M_{i,j-1}-\alpha \\ \end{array} \right.\\ \end{aligned}  $$

Termination:

3 $$ Result=max \left\{ \begin{array}{l} \max_{\{1 \leq i \leq m \}} M_{i,n}\\ \max_{\{ {1 \leq j \leq n} \}} M_{m,j}\\ \end{array} \right.  $$

where m and n are the length of R1 and R2, respectively. In these equations, *M*_*i*,*j*_ represents the optimal alignment score of two subsequences *R*1[1]...*R*1[*i*] and *R*2[1]...*R*2[*j*], while *I*_*i*,*j*_ and *D*_*i*,*j*_ represent the optimal alignment score of two subsequences *R*1[1]...*R*1[*i*] and *R*2[1]...*R*2[*j*] with *R*2[*j*] aligned to a gap and *R*1[*i*] aligned to a gap, respectively. Here, the semi-global alignment uses an affine gap penalty model to calculate gap penalties, in which *α* and *β* are the gap open penalty and the gap extension penalty, respectively. *sbt* is the score of a match or mismatch. As shown by Eq. 1, the penalties of gaps at the start and end of two sequences are neglected. As shown by Eq. 3, the optimal alignment score of the semi-global alignment in GATK HC is the greatest value of the elements in the last row and the last column of the matrix M.

These equations indicate that the computation complexity of the modified Needleman-Wunsch algorithm is *O*(*mn*), which makes the execution time increase quadratically with the sequence length. Usually, the algorithm is implemented using dynamic programming which solves three two dimensional matrices. According to the equations, *M*_*i*,*j*_, *I*_*i*,*j*_ and *D*_*i*,*j*_ only depend on the up-left, up and left neighbor elements, which implies that the elements on the same anti-diagonal can be computed in parallel. Thus, a method employed by many researchers to reduce the execution time is to exploit this inherent parallelism in the algorithm.

**Fig. 2:**
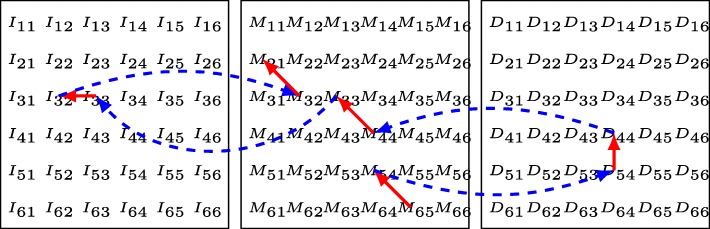
An example of backtracking an optimal alignment. The backtracking of an optimal alignment starts from *M*_6,5_ (central matrix in the figure), passes through *M*_5,4_, jumps to *D*_5,4_ (right matrix in the figure), passes through *D*_4,4_, jumps to *M*_4,4_, passes through *M*_3,3_, jumps to *I*_3,3_ (left matrix in the figure), passes through *I*_3,2_, jumps to *M*_3,2_ and ends at *M*_2,1_. The optimal alignment retrieved is “MDMIM”

If the alignment only needs to find the optimal alignment score of two sequences, the dynamic programming kernel can be calculated in linear space. Otherwise, the alignment with affine gap penalties generally uses three backtracking matrices to store the scores or alignment moves calculated by the dynamic programming kernel. The optimal alignment traceback starts from the position of the element with the optimal alignment score until reaching any element in the first row or the first column of the backtracking matrices, which is calculated in linear time. Figure 2 presents an example of backtracking an optimal alignment based on the score matrices.

### Cigar format

In GATK HC, the goal is to get the optimal semi-global alignments, which are represented in the CIGAR format [18], and POS. CIGAR is a string including one or more number-character pair(s). The character, including ‘M’, ‘I’, ‘D’, ‘N’, ‘S’, ‘H’, ‘P’, ‘=’ and ‘X’, defines an operation explaining how the base in R2 aligns to the base in R1. Table 1 shows the CIGAR operations used in GATK HC. The number defines the length of the consecutive operations. POS is 0-based left most position of the first matching base of R1, which indicates the position of R1 where the alignment starts.

**Table 1:** CIGAR operations used in GATK HC

Operation	Description
M	Match/mismatch
I	Insertion (gap in R1)
D	Deletion (gap in R2)
S	Soft clipping (base at the beginning or the end of R2 but not in R1)

Take the alignment in Fig. 1 for example. The CIGAR representation of the alignment is “3M2D1M2I2M1S” and POS of the alignment is 1.

### GPU architecture

Modern GPUs are widely used to accelerate computationally intensive algorithms. A GPU consists of thousands of small cores capable of executing one thread at a time. On NVIDIA GPUs, threads are grouped into *blocks* and these blocks are grouped into *grids*. Furthermore, consecutive threads in the same block are grouped into *warps*. The size of a warp is usually 32. The memory hierarchy includes registers, shared memory, global memory, cache and so on. Each thread is assigned a set of registers. The shared memory is accessed by all threads in a block. Using the shared memory, the threads in a block can exchange data at a very fast rate. The global memory is accessed by all the threads on the GPU. The latency of the global memory access is high since it resides on the device DRAM. If the data accessed by each thread in the same warp are stored at consecutive addresses, the global memory accesses of these threads can be coalesced. Usually, the width of one global memory access is 128 bytes. If the global memory accesses of threads in a warp are coalesced, there will be only one global memory access when the data accessed by each thread is not more than 4 bytes. Otherwise, there would be 32 sequential global memory accesses in the worst-case situation.

### Semi-global alignment in GATK HC

#### Implementation of alignment in GATK HC

In GATK HC, the semi-global pairwise alignment is performed in two stages.

The implementation of the first stage is realized with a two-layer loop, which results in the *O*(*mn*) computational complexity. The results of the first stage are two matrices: the score matrix *sw*, which stores matrix *M*, and the backtracking matrix *btrack*. In *btrack*, the value of each element can be classified into three kinds, which is defined as follows:

- >0 - indicates a deletion and the length of the consecutive deletion(s) is the value of the element
- =0 - indicates a match or mismatch and the length of the consecutive match(es)/mismatch(es) is increased by 1
- <0 - indicates an insertion and the length of the consecutive insertion(s) is the absolute value of the element

The absolute values of the elements in the backtracking matrix are calculated by recording the length of the consecutive deletion(s) and consecutive insertion(s) when calculating the score matrix.

The implementation of the second stage is to calculate the optimal alignment in CIGAR format and POS. The score matrix sw is first used to find the optimal alignment score and the backtracking matrix *btrack* is then used to obtain the optimal alignment and POS. The optimal alignment is calculated in linear time. The backtracking matrix in GATK HC is helpful during backtracking. It is much easier to identify the next move compared with other methods since it does not need to jump among several backtracking matrices (shown in Fig. 1) or calculate the next move based on the current move [12]. Moreover, the lengths of the consecutive deletion(s) and consecutive insertion(s) are given by the element of the *btrack* matrix. However, the length of the consecutive match(es)/mismatch(es) is not given, which is increased by one instead.

#### Data analysis

In GATK HC, the semi-global alignment is performed in three situations:

1. Align the reference path with the dangling path to recover dangling branches for the local assembly.
2. Align the read with the assembled haplotype.
3. Align the assembled haplotype with the reference to decide whether the assembled haplotype satisfied the defined requirements.

We profiled GPU-based GATK HC [3] with a typical workload (Chromosome 10 of the whole human genome dataset G15512.HCC1954.1 [19]) to investigate which situation is most time-consuming. The profiling results in single-threaded mode are shown in Table 2, which specifies the relative execution time and the number of the semi-global alignments in each situation. As shown in the table, the execution time of all the semi-global alignment accounts for 34.5% of the total execution time. Moreover, situation 2 and 3 consumes around 100% of the semi-global alignment execution time and the execution time in situation 1 is negligible. However, although the number of semi-global alignments in situation 2 is much larger than that in situation 3, the execution time in situation 2 is smaller than that in situation 3.

**Table 2:** Execution time of the semi-global alignment in three situations of GATK HC

Situation	Number of alignments	Execution time
1	3529	0.03%
2	850376	14.58%
3	54802	19.89%
Total	908707	34.5%

**Fig. 3:**
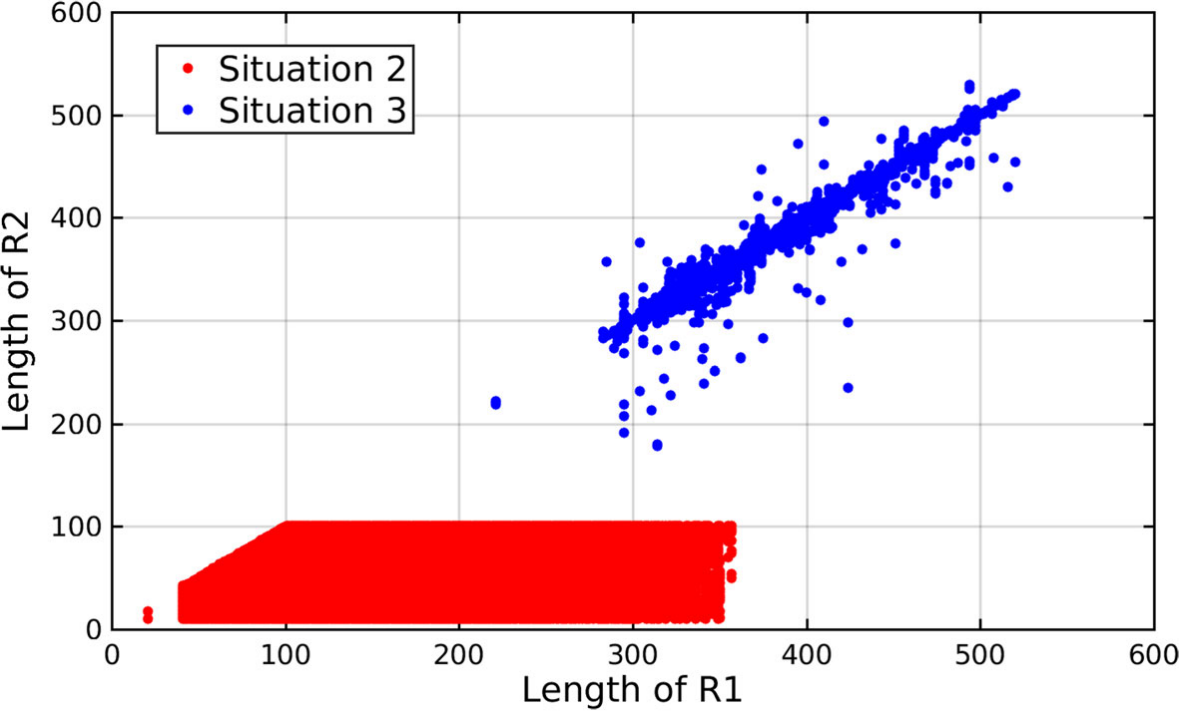
Lengths of sequence pairs in situation 2 and 3. The lengths of the sequence pairs in situation 2 (40 ∼350) are shorter than those in situation 3 (300 ∼520)

We then analyzed the lengths of the sequence pairs in situation 2 and 3. In situation 2, let R1 be the assembled haplotype and R2 be the read. In situation 3, let R1 be the assembled haplotype and R2 be the reference. Figure 3 shows a scatter plot of the lengths of the sequence pairs in these two situations. As shown in Fig. 3, the lengths of the sequence pairs in situation 2 (40 ∼350) are shorter than those in situation 3 (300 ∼520). Since the computation complexity is *O*(*mn*), the execution time of each semi-global alignment in situation 3 is bigger than that in situation 2. This explains why the total execution time of situation 3 is bigger than that of situation 2, which is shown in Table 2. Moreover, in situation 2, the length of R2 (the read) is shorter than the length of R1 (the assembled haplotype).

**Fig. 4:**
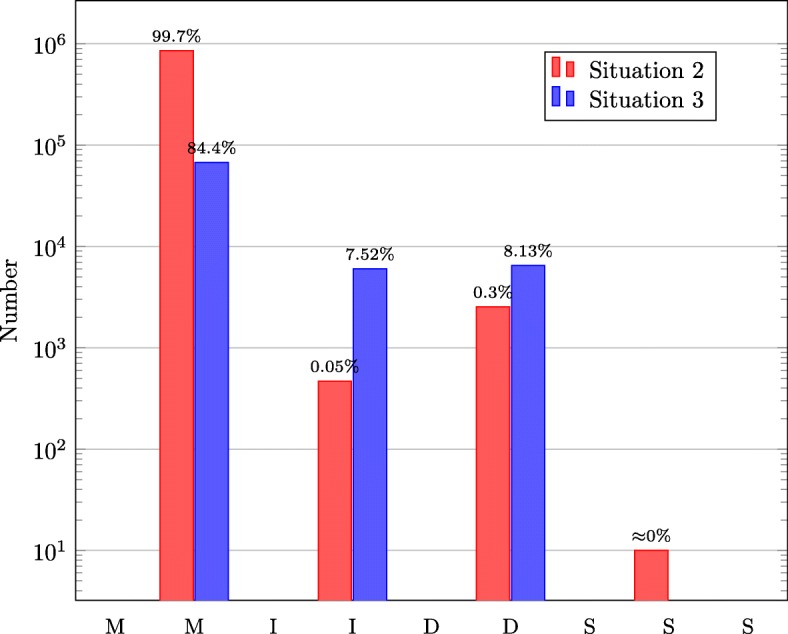
Numbers of M/I/D/S in situation 2 and 3. The number of *M* operations is the largest. Especially in situation 2, the number of *M* operations accounts for 99.65% of the total operations

In addition, we investigated the optimal alignments achieved in situation 2 and 3 and added up the number of M/I/D/S operations in each optimal alignment. Figure 4 shows that the number of *M* operations is the largest. Especially in situation 2, the number of *M* operations accounts for 99.65% of the total operations. Moreover, we found that most of *M* operations are consecutive in each optimal alignment. However, the length of the consecutive match(es)/mismatch(es) is increased by one during the optimal alignment retrieval.

Hence, although the computation complexity of the optimal alignment is linear, most of its execution time is used to calculate the length of the consecutive match(es)/mismatch(es).

**Fig. 5:**
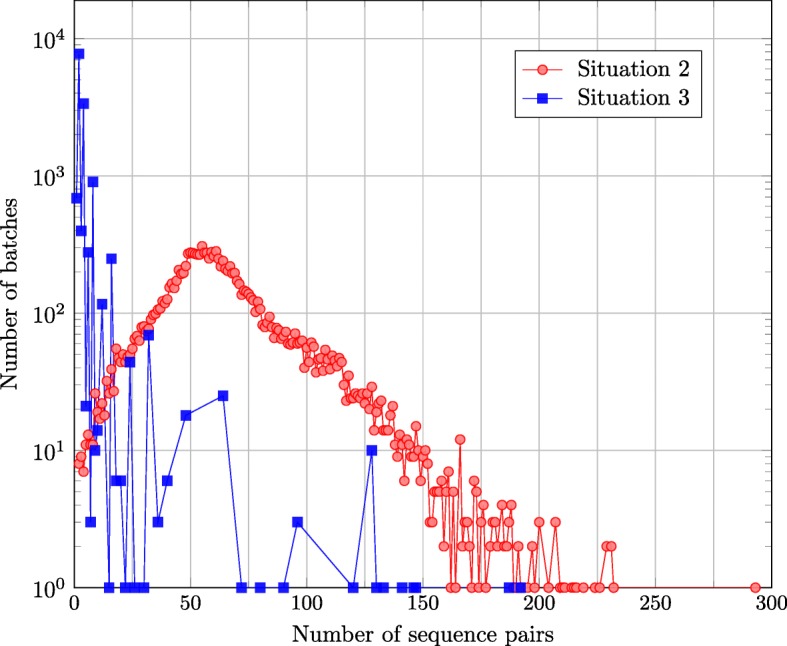
Numbers of batches including different number of sequence pairs in situation 2 and 3. The biggest number of sequence pairs in all the batches in situation 2 and 3 are 293 and 192, respectively. Furthermore, the majority of batches in situation 2 include 25 ∼125 of sequence pairs while the majority of batches in situation 3 include 1 ∼8 of sequence pairs

We last studied the source code of GATK HC version 3.7 and found that the semi-global alignments in situation 2 and 3 can be grouped into many batches without big modifications of the source code. Each batch consists of many semi-global alignments of sequence pairs. The numbers of batches in situation 2 and 3 are 13,142 and 13,977, respectively. Figure 5 shows the number of sequence pairs of each batch in situation 2 and 3. The biggest number of sequence pairs in all the batches in situation 2 and 3 are 293 and 192, respectively. Furthermore, the majority of batches in situation 2 include 25 ∼125 of sequence pairs while the majority of batches in situation 3 include 1 ∼8 of sequence pairs.

### Implementation on GPUs

The implementation of the semi-global pairwise alignment for GATK HC on GPUs is performed in two stages. In the first stage, it performs the modified Needleman-Wunsch algorithm in order to obtain the backtracking matrix and the position of the optimal alignment score. In the second stage, it retrieves the backtracking matrix in order to obtain the optimal alignment in CIGAR format and POS.


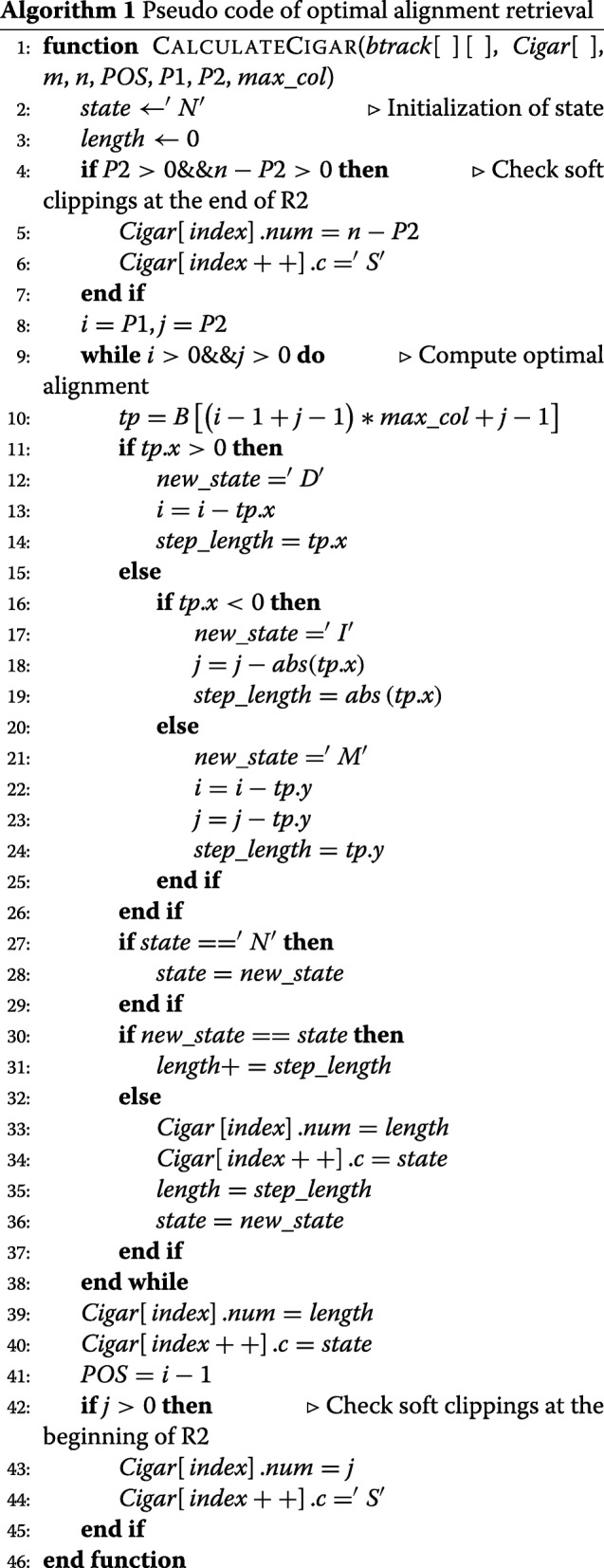


#### First stage implementation

##### Intra-task parallelization

**Fig. 6:**
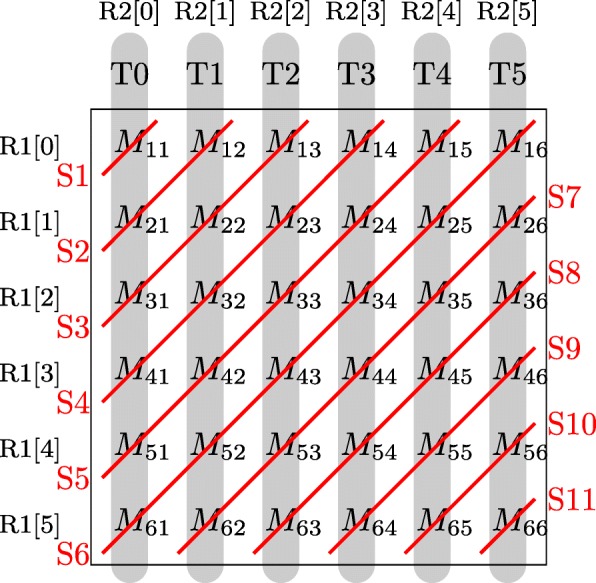
Calculation of matrix M on GPUs

As mentioned in “Data analysis” subsection, the number of sequence pairs in each batch is less than 300. In order to effectively use the resources on GPUs, the intra-task parallelization model is employed to implement the modified Needleman-Wunsch algorithm on GPUs. For the implementation on GPUs, the elements on the same anti-diagonal of the score matrix M, I and D and backtracking matrix are calculated in parallel, reducing the computational complexity to *O*(*m*+*n*). Figure 6 shows the calculation of matrix *M* as an example to explain the implementation. Let R1 and R2 be the two sequences. There are in total 6 threads in the block and the size of matrix M is 6×6. At each step, the elements on an anti-diagonal are calculated in parallel and every element is calculated by one thread. For example, at step 5 (S5), *M*_5,1_, *M*_4,2_, *M*_3,3_, *M*_2,4_ and *M*_1,5_, which are on the same anti-diagonal, are calculated by thread 0 (T0), thread 1 (T1), thread 2 (T2), thread 3 (T3) and thread 4 (T4), respectively. These elements are then used in the next step to calculate the elements on the next anti-diagonal. Moreover, each thread is responsible to calculate the elements in one column of matrix *M*. For example, the elements in the second column are calculated by thread 1 (T1). The goal of the first stage is to obtain the backtracking matrix and the position of the optimal alignment score. Therefore, elements of the score matrix *M*, *I* and *D* are not stored. Instead, two vectors in the shared memory and three registers of each thread are used to store the intermediate results of the three score matrices. During the calculation of elements of the last column and the last row of matrix M, the optimal alignment score and its position are obtained. However, the drawback of the implementation is that some threads remain idle at the beginning or at the end of the calculation procedure, resulting in low thread utilization. If the length of R2 is smaller than the number of threads in a block, the execution is similar to Fig. 6 while some threads would remain idle during the whole calculation procedure. If the length of R2 is bigger than the number of threads in a block, there are two solutions to deal with it. One is to increase the size of a block until the number of threads in a block is equal to or bigger than the length of R2. The other is to divide the calculation into several passes. In each pass, the execution is similar to Fig. 6. Three vectors in the global memory are used to store the intermediate results produced by the last thread of each pass, which would be used in the next pass. The advantage of the second solution is that it increases efficiency by reducing idle percentage of threads during the calculation procedure while its disadvantage is that it increases global memory accesses.

##### Recording the length of consecutive match(es)/mismatch(es)

Besides recording the length of the consecutive deletion(es)/insertion(es), we also record the length of the consecutive match(es)/mismatch(es) in the first stage. The backtracking matrix on GPUs is stored in a *short*2 matrix. Each element of the matrix has two values, which are x and y. The value of x and y are defined as follows:

- *x*>0 - indicates a deletion and the length of the consecutive deletion(s) is the value of the element
- *x*=0 - indicates a match or mismatch and the length of the consecutive match(es)/mismatch(es) is y.
- *x*<0 - indicates an insertion and the length of the consecutive insertion(s) is the absolute value of the element

The data type of x and y is *short*, of which the minimum value and maximum value are −32768 and 32767, respectively. The absolute values of the minimum value and maximum value are bigger than the theoretical maximum length of the consecutive operations, which is the length of R1 or R2. In order to calculate the backtracking matrix, a *short*2 vector in the shared memory and two registers of each thread are used.

**Fig. 7:**
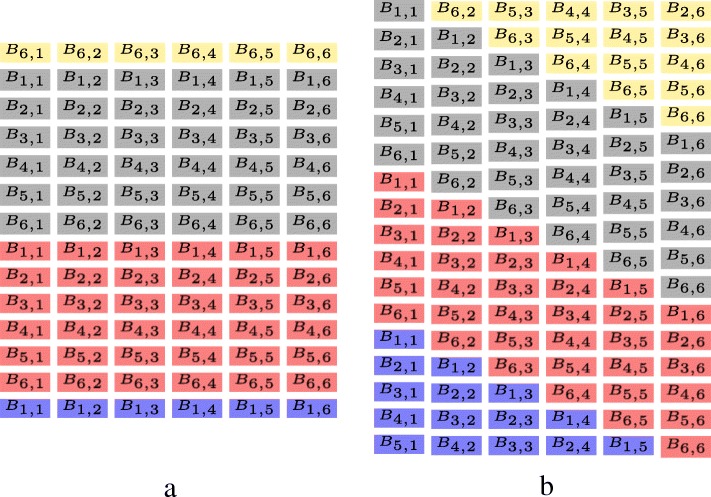
Layout of the backtracking matrix in the global memory. Elements of different backtracking matrices are marked with different colors. **a** is the backtracking matrices stored in the row-major data format. **b** is the backtracking matrices stored in the diagonal-major data format

Moreover, Since the backtracking matrix will be used in the next stage and the shared memory is not big enough to store it, the backtracking matrix is stored in the global memory. Similar to calculation of the matrix *M* shown in Fig. 6, elements of the backtracking matrix are calculated in anti-diagonal order. Thus, the backtracking matrices are stored in the diagonal-major data format (Fig. 7b), which is proposed in [20], instead of the row-major data format (Fig. 7a) to avoid non-coalesced global memory accesses of 32 threads in a warp and reduce global memory accesses.

#### Second stage implementation

In the second stage, we use the backtracking matrix *btrack* to obtain the optimal alignment and POS. Algorithm 1 presents the pseudo code of the optimal alignment retrieval on GPUs. *P*1 and *P*2 describe the position of the optimal alignment score. Algorithm 1 first checks whether there are soft clippings at the end of R2, and then computes the optimal alignment in a *while* loop. At the end, it checks whether there are soft clippings at the beginning of R2. The backtracking starts from (*P*1,*P*2) and finishes when *i*≤0 or *j*≤0, which is calculated in linear time. POS is the value of (*i*−1) at the end of the *while* loop. In addition, the position of each element in the backtracking matrix is calculated by *i*, *j* and *max*_*col*, as shown in the 9th line in Algorithm 1. *max*_*col* is the column size of the maximum backtracking matrix of all sequence pairs.

The length of the deletion, insertion and match/mismatch is given by the value of an element of the backtracking matrix, as shown in the 13th, 18th and 23rd line, respectively. This reduces the global memory accesses used to calculate the length of the operations.

## Results

All the experiments are performed on IBM Power System S823L (82478-42L), which has 2 IBM Power8 processors (10 cores each) running at 3.6 GHz, 256 GB of DDR3 memory, and an NVIDIA Tesla K40 card. The NVIDIA Tesla K40 card has 2880 cores that run at up to 745 MHz and has a CUDA compute capability of 3.5.

We first compare the performance of the GPU-based semi-global alignment implementation with different techniques using the synthetic datasets. The synthetic datasets are created based on the output of Wgsim [21] with default parameters. We then compare the performance of GPU-based semi-global alignment implementation with gpu-pairAlign implementation using synthetic datasets. Next, we compare the performance of GPU-based semi-global alignment implementation with CPU-based implementation using synthetic and real datasets. We last integrate the GPU-based semi-global alignment implementation into GATK HC 3.7 and compare the overall performance.

Throughput is used as a performance metric of the first stage of the GPU-based implementation, which is measured by giga cell updates per second (GCUPS). Note that it is not fair to compare the throughput of the first stage of the semi-global alignment with traceback with that of the score-only alignments since the former needs to store backtracking matrices in the global memory.

### Performance comparison of multi-pass

There are two solutions to implement the first stage of the semi-global alignment on GPUs if the length of R2 is bigger than the number of threads in a block. We realized these two solutions and used different synthetic datasets to compare the performance of the two solutions.

**Fig. 8:**
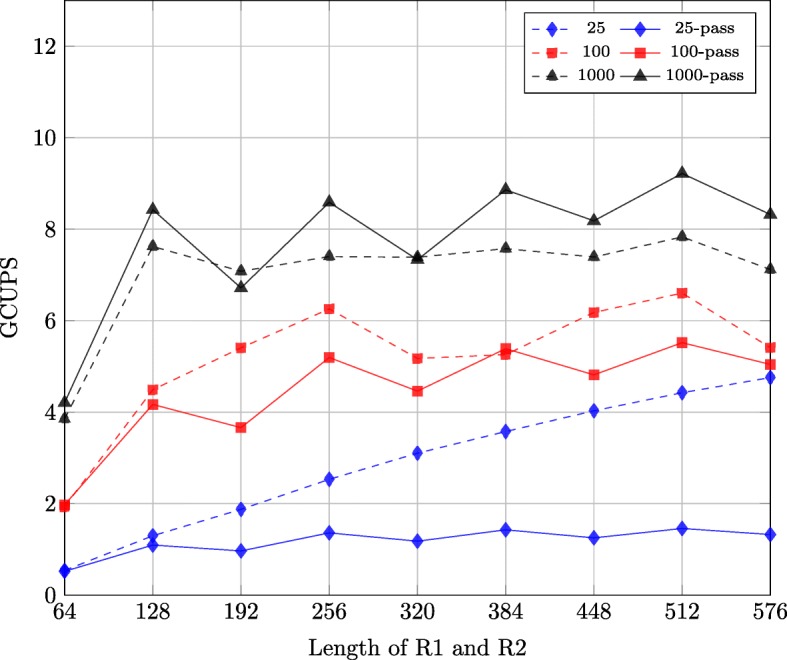
Performance comparison of implementations for two solutions on synthetic datasets

Figure 8 shows the performance of the two solutions with different synthetic datasets. There are 9 datasets each with a different length of R1/R2, namely: 64, 128, 192, 256, 320, 384, 448, 512 and 576. In each dataset, the lengths of R1 and R2 are the same. The number of sequence pairs in the 9 datasets is 25, 100 and 1000.

For the first solution, which is to increase the block size, there are in total 9 implementations for 9 datasets. The differences of these implementations are the block size and the sizes of vectors in the shared memory which store the intermediate results. For the second solution, which employs multi-pass, there is 1 implementation with block size of 128.

As shown by Fig. 8, the throughput of the first solution is higher than that of the second solution when the number of sequence pairs of the datasets is 25 and 100. However, when the number of sequence pairs of the datasets is 1000, the throughput of the second solution is higher in most cases. This is because the efficiency of the implementations for the first solution is smaller than that of the implementation for the second solution and the advantage of the second solution overweighs its disadvantage when the number of sequence pairs of the dataset is big. Thus, we can choose the implementation of these two solutions based on the number of sequence pairs of the dataset.

### Performance comparison of recording match/mismatch lengths

In this section, we analyze the impact of recording the length of consecutive matches/mismatches on the performance of the second stage of the alignment on GPUs. We realized two implementations. The first implementation is our approach shown in Algorithm 1 in which the length of consecutive matches/mismatches is recorded in the backtrack matrix. The second implementation is similar to Algorithm 1 except that the length of M is increased by one and the coordinates (*i*, *j*) of M are decreased by 1. The backtracking matrices are produced by 9 implementations for the first solution using 9 synthetic datasets. Here, the synthetic datasets are not based on the output of Wgsim since we consider the best case, in which only many M operations exist in the optimal alignment. The lengths of R1/R2 in the 9 synthetic datasets are 64, 128, 192, 256, 320, 384, 448, 512 and 576. The number of sequence pairs in the 9 datasets is 100.

**Fig. 9:**
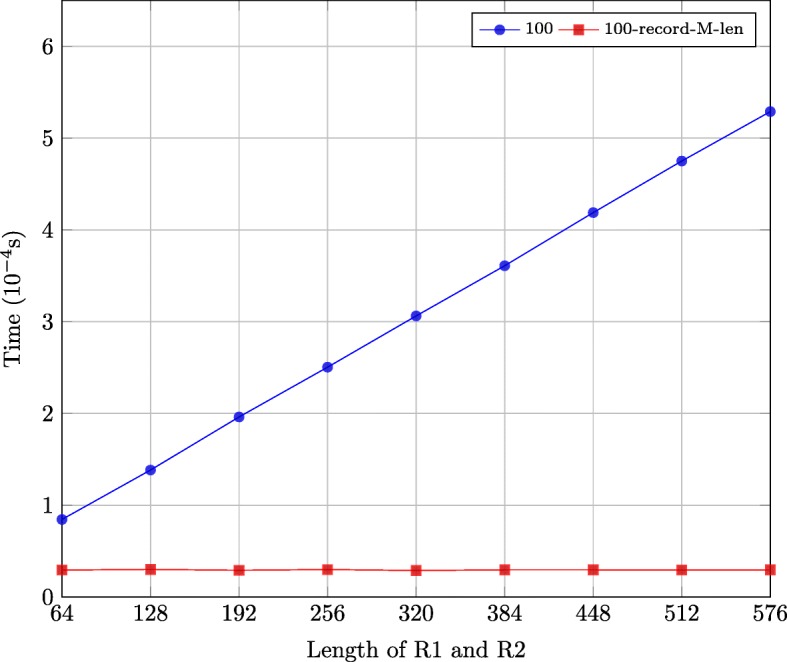
Execution time of GPU-based optimal alignment backtracking implementations (not) recording match/mismatch lengths on synthetic datasets

Figure 9 shows the execution time of the two implementations. The implementation which records match/mismatch lengths is faster. Moreover, its execution time remains nearly constant with increasing length of R1 and R2 as it only requires a single global memory access per R1 and R2 pair. The execution time of the implementation without recording match/mismatch lengths increases linearly with the length of R1 and R2. This is because the number of global memory accesses increases linearly with the number of *M* operations in the optimal alignment, which in turn increases linearly with the length of R1 and R2.

### Performance comparison with gpu-pairAlign

As mentioned in “Background” section, there are two methods to implement the second stage on GPUs: the method based on the Myers-Miller algorithm and the method based on backtracking matrices. The method based on the Myers-Miller algorithm is only suitable for the pairwise alignment of very long DNA and protein sequences. Thus, we compared our implementation with gpu-pairAlign [12], which uses backtracking matrices to obtain the optimal alignments. gpu-pairAlign is designed to perform alignment of every given sequence pair on GPUs, especially for protein sequence pairs. It includes algorithms for global alignment, semi-global alignment and local alignment. We compare with its semi-global alignment algorithm. The semi-global alignment algorithm of gpu-pairAlign is also performed in two stages: the optimal alignment score and the backtracking matrices are computed in the first stage; the backtracking is performed in the second stage.

There are two main differences between the gpu-pairAlign implementation and our implementation: (1) In the first stage, our implementation employs the intra-task parallelization model, while the gpu-pairAlign implementation employs the inter-task parallelization model; (2) The backtracking matrix of our implementation is a short2 matrix, elements of which are the length of consecutive deletion(es), insertion(es) and match(es)/mismatch(es), while the backtracking matrices of the gpu-pairAlign implementation are four Boolean matrices, elements of which indicate the proper direction of backtracking moves.

We modified the gpu-pairAlign implementation to make it to deal with the data produced by GATK HC: (1) Since the input data of our implementation is a set of sequence pairs instead of a set of sequences, the way in which the gpu-pairAlign implementation handles input data is modified; (2) Integer arrays are used to store the intermediate results instead of short arrays since the intermediate results are bigger than the maximum value of the short data type; (3) The alignments are modified to be represented using the CIGAR format and POS.

**Fig. 10:**
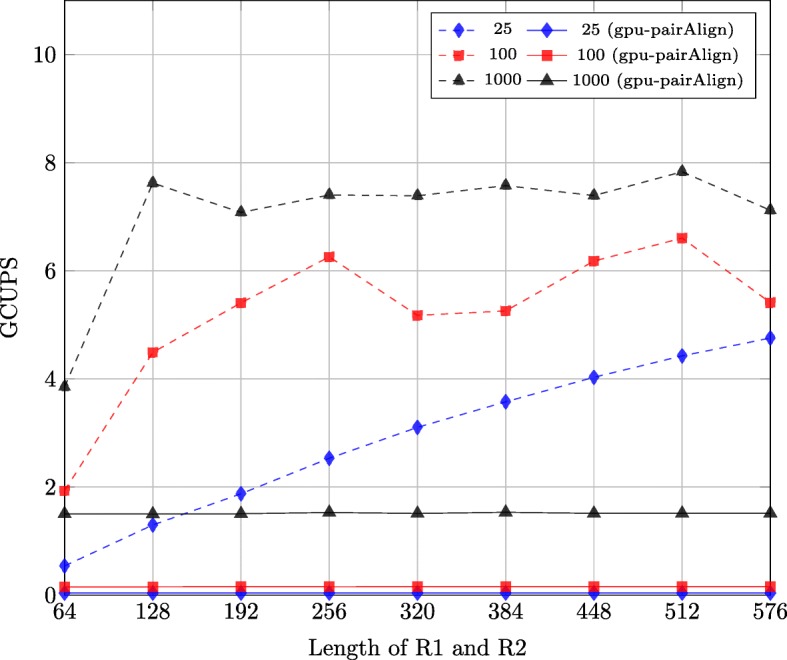
Performance comparison of the first stage of two implementations on synthetic datasets

We first used the synthetic datasets described in “Performance comparison of multi-pass” subsection to compare the performance of the first stage of the two implementations, which is shown in Fig. 10. The performance of gpu-pairAlign implementation is much smaller than our implementation. The main reason is that when the size of the synthetic datasets is small, the resource on the GPU cannot be fully utilized for the inter-task parallelization model. The second reason is that the intermediate results are stored in integer arrays, which increases the size of shared memory of each block and the number of global memory accesses.

**Fig. 11:**
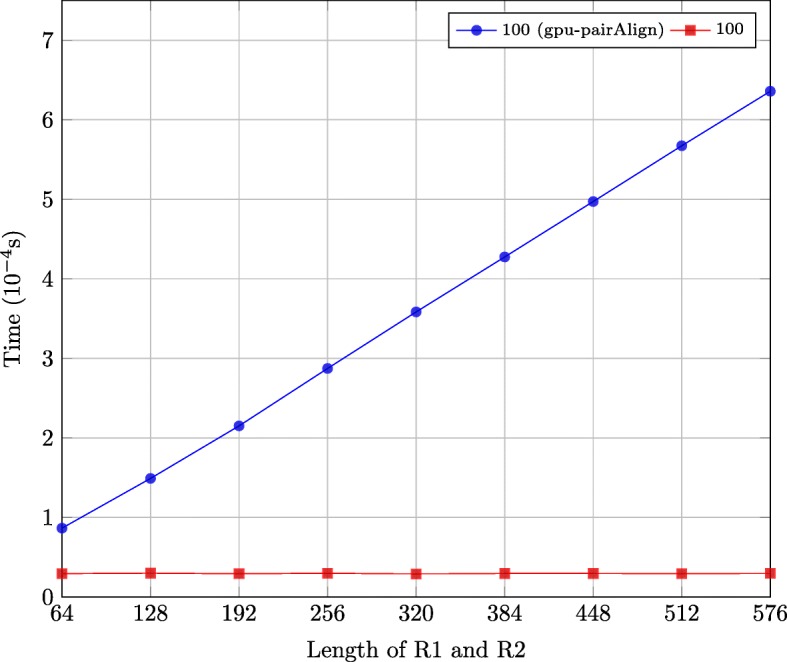
Execution time of the second stage of two implementations on synthetic datasets

We then compared the performance of the second stage of the two implementations using the synthetic datasets described in “Performance comparison of recording match/mismatch lengths” subsection, which is shown in Fig. 11. The execution time of the second stage of our implementation remains nearly constant when the length of R1 and R2 increases, while the execution time of the second stage of the gpu-pairAlign implementation increases linearly with the length of R1 and R2. Although the gpu-pairAlign implementation reduces the global memory space by using four Boolean matrices, it still needs to calculate each move one by one, which is avoided in our implementation through storing the length of consecutive deletion(es), insertion(es) and match(es)/mismatch(es).

### Performance comparison with CPU-based implementation

In this section, we compare the performance of our GPU-based semi-global alignment with traceback implementation with the CPU-based implementation using synthetic and real datasets. We used the first solution which increases the block size when the length of R2 is bigger than the block size and records the length of consecutive matches/mismatches. The CPU-based implementation is written in the C++ programming language and compiled with gcc O3 optimization, running on one Power8 core. The real datasets are produced by using a typical workload (Chromosome 10 of the whole human genome dataset G15512.HCC1954.1).

**Fig. 12:**
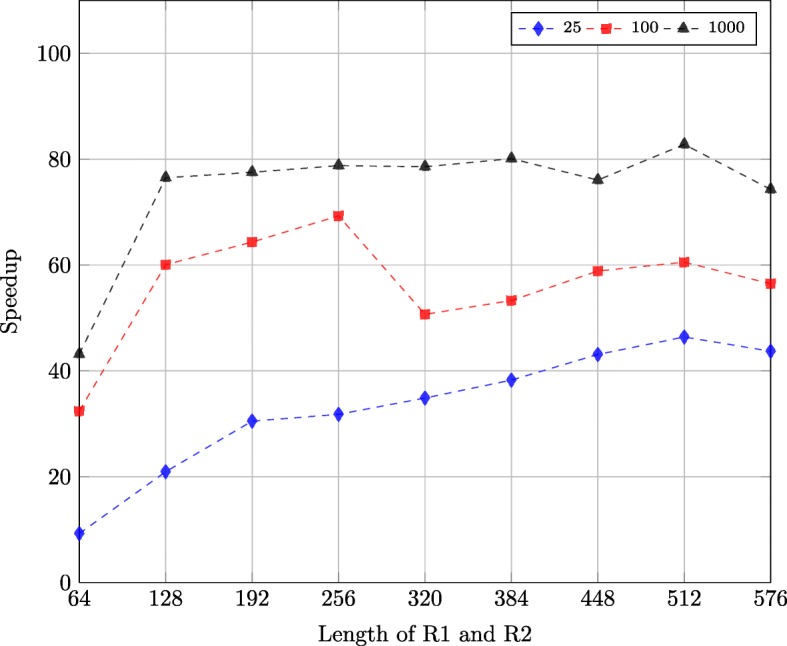
Speedup of the GPU-based implementations compared with CPU-based implementation on synthetic datasets

Figure 12 shows the speedup of the GPU-based implementations compared with the CPU-based implementation using the synthetic datasets described in “Performance comparison of multi-pass” subsection. There are in total 9 GPU-based implementations for 9 datasets, block size of which are 64, 128, 192, 256, 320, 384, 448, 512 and 576. The GPU-based implementations is up to 80x faster than the CPU-based implementation. Moreover, the speedup of the datasets with 1000 sequence pairs is bigger than the speedup of the datasets with 25 and 100 sequence pairs.

Table 3 shows the execution time of GPU-based implementations with the real datasets. As shown by Fig. 3, the length of R2 in situation 2 is 40 ∼120 and the length of R2 in situation 3 is 300 ∼520. Thus, we used two GPU-based implementations with block size of 128 and 576 to execute the real datasets produced in situation 2 and 3, respectively. The GPU-based implementation of situation 2 is 14.14x faster than the CPU-based implementation, while the GPU-based implementation of situation 3 is 4.89x faster than the CPU-based implementation. The throughput of the first stage of the GPU-based implementation for situation 2 is 1.86 GCUPS, while that for situation 3 is 0.64 GCUPS. The throughput of situation 3 is much smaller than the throughput for the synthetic datasets with size 25. This is because the number of sequence pairs of batches in situation 3 is extremely small (1∼8 in most cases).

**Table 3:** Performance of GPU-based implementations on real datasets

	Throughput (GCUPS)	GPU (sec)	CPU (sec)	Speedup
Stage 1 of S2	1.86	2.32	43.93	18.94x
Stage 2 of S2	-	0.14	0.62	4.43x
**Overall of S2**	-	3.15	44.55	14.14x
Stage 1 of S3	0.64	10.20	53.29	5.22x
Stage 2 of S3	-	0.09	0.17	1.89x
**Overall of S3**	-	10.93	53.46	4.89x

S2 and S3 stand for situation 2 and 3, respectively. “Overall of S2” and “Overall of S3” represent the overall GPU execution time? CPU execution time and speedup of situation 2 and 3, respectively

### Integration into GATK HC

The two GPU-based implementations with block size of 128 and 576 are integrated into GATK 3.7 to accelerate the semi-global alignment with traceback of situation 2 and situation 3, respectively. The GATK HC implementation with both GPU-based pair-HMMs forward algorithm and GPU-based semi-global alignment with traceback is compared with other two GATK HC implementations: GATK HC (referred to as baseline), which is downloaded from the GATK website, and GATK HC with only GPU-based pair-HMMs forward algorithm. The dataset is Chromosome 10 of the whole human genome dataset (G15512.HCC1954.1). All the GATK HC implementations are performed in single thread mode.

Table 4 shows the overall execution time of these three implementations. The implementation with both GPU-based pair-HMMs forward algorithm and GPU-based semi-global alignment with traceback is 2.30x faster than the baseline implementation. Moreover, it is 1.34x faster than the implementation with only GPU-based pair-HMMs forward algorithm.

**Table 4:** Execution time of GATK HC implementations

	Total time (s)	Speedup
Baseline	8034.05	-
GPU (only pair-HMMs)	4687.08	1.71x
GPU (pair-HMMs + semi-global alignment with traceback)	3490.70	2.30x

Note that the number of sequence pairs of each batch produced by GATK HC is small, leading to under utilization of the GPU resources. It is better to launch multiple GATK HC processes at the same time to fully utilize the GPU resources.

## Conclusion

This paper presents an implementation of the semi-global alignment with traceback on GPUs to improve the performance of GATK HC. Semi-global alignment with traceback has two stages: in the first stage, a backtracking matrix is computed; in the second stage, the optimal alignment is calculated using the backtracking matrix. Based on the characteristics of the semi-global alignment with traceback in GATK HC, the intra-task parallelization model is chosen. The first stage of our GPU implementation is up to 18.94x faster than CPU. Moreover, our GPU implementation also records the length of consecutive matches/mismatches in addition to lengths of consecutive insertions and deletions as in the CPU implementation. This helps to reduce global memory accesses and provides a speedup of up to 4.43x in the second stage. Experimental results show that our alignment kernel with traceback is up to 80x and 14.14x faster than its CPU counterpart with synthetic datasets and real datasets, respectively. The GATK HC implementation with both GPU-based pair-HMMs forward algorithm and GPU-based semi-global alignment with traceback is 2.30x faster than the baseline GATK HC. It is 1.34x faster than the GATK HC implementation with only GPU-based pair-HMMs forward algorithm.

## Abbreviations

HC: HaplotypeCaller
NGS: Next Generation Sequencing
